# The mitochondrial genome of *Leptopeza flavipes* (*Diptera*: Empididae)

**DOI:** 10.1080/23802359.2020.1788458

**Published:** 2020-07-23

**Authors:** Yue Liu, Mengqing Wang, NaiZhong Chen, Ding Yang

**Affiliations:** a College of Plant Protection, China Agricultural University, Beijing, China; b Institute of Plant Protection, Chinese Academy of Agricultural Sciences, Beijing, China; c Institute of Equipment Technology, Chinese Academy of Inspection and Quarantine, Beijing, China

**Keywords:** Mitochondrial genome, Ocydromiinae, phylogenetics

## Abstract

The dance fly *Leptopeza flavipes* belongs to the subfamily Ocydromiinae of Empididae. The mitogenome (GenBank accession number: MT610901) of *L. flavipes* was sequenced, the new representative of the mitogenome of the subfamily. The nearly complete mitogenome is 15,267 bp totally, consisting of 13 protein-coding genes, 2 rRNAs and 22 transfer RNAs. All genes have similar locations and strands with that of other published species of Empididae. The nucleotide composition biases toward A and T, which together made up 78.1％of the entirety. Bayesian inference analysis strongly supported the monophyly of Empidoidea, Empididae and Dolichopodidae. It is clear that the phylogenetic relationship within Empidoidea: (Dolichopodinae + Neurigoninae) + ((Empidinae + (Trichopezinae + Oreogetoninae)) + Ocydromiinae) in this study.

The dance flies or Empididae are one of the largest groups in Brachycera (Diptera) with over 5,000 known species worldwide, and belong to the superfamily Empidoidea. The adults and larvae are predacious, including some important predators of some pest insects such as aphises, psyllids, whiteflies, flies, and mites, except for adults of some species being flower visitors (Yang et al. 2007).

The specimens of *Leptopeza flavipes* used for this study were collected in St Petersburg City of Russia by Igor Shamshev and identified by Igor Shamshev. Specimens are deposited in the Entomological Museum of China Agricultural University (CAU) with the accession number is CAUYD3050 (Room 2005, Plant Protection Building, West Campus, China Agricultural University). The total genomic DNA was extracted from the whole body (except head) of the specimen using the QIAamp DNA Blood Mini Kit (Qiagen, Germany) and stored at −20 °C until needed. The mitogenome was sequenced in BeiRuiHeKang biotechnology company used NGS. The nearly complete mitogenome of *L. flavipes* is 15,267 bp (GenBank accession number: MT610901). It encoded 13 PCGs, 22 tRNA genes and 2 rRNA genes and were similar with related reports before (Hou et al. 2019; Qilemoge et al. 2019; Yang et al. 2019; Qilemoge et al. 2020). All genes have the similar locations and strands with that of other published Empididae species. The nucleotide composition of the mitogenome was biased toward A and T, with 78.1% of A + T content (A = 39.6%, T = 38.5%, C = 12.8%, G = 9.1%). The A + T content of PCGs, tRNAs, and rRNAs is 76.5%, 79.1%, and 82.4% respectively. The total length of all 13 PCGs of *L. flavipes* is 11,256 bp. Three PCGs (*NAD2*, *NAD5, NAD6*) initiated with ATT codons, and seven PCGs (*COII*, *COIII*, *ATP6*, *NAD3*, *NAD4*, *NAD4L* and *CYTB*) initiated with ATG codons, and *COI* initiated with TTG as a start codon, and *NAD1* and ATP8 initiated with ATA as a start codon. All thirteen PCGs used the typical termination codons TAA in *L. flavipes.*

Phylogenetic analysis was performed based on the nucleotide sequences of 13 PCGs from 10 Diptera species. Bayesian (BI) analysis generated the phylogenetic tree topologies based on the PCGs matrices (Figure 1). The phylogenetic result shows that the monophyly of Empidoidea, Dolichopodidae and Empididae were strongly supported, which is consistent with the phylogenetic result of the previous research (Wang et al. 2016). The monophyletic Dolichopodidae that contains Dolichopodinae and Neurigoninae was assigned to the sister group to the clade of Empididae that consists of Empidinae, Trichopezinae, Oreogetoninae and Ocydromiinae in this study. It is clear that the phylogenetic relationship within Empidoidea: (Dolichopodinae + Neurigoninae) + ((Empidinae + (Trichopezinae + Oreogetoninae)) + Ocydromiinae) in this study. The mitogenome of *L. flavipes* could provide the important information for the further studies of Empidoidea phylogeny.

**Figure 1.**
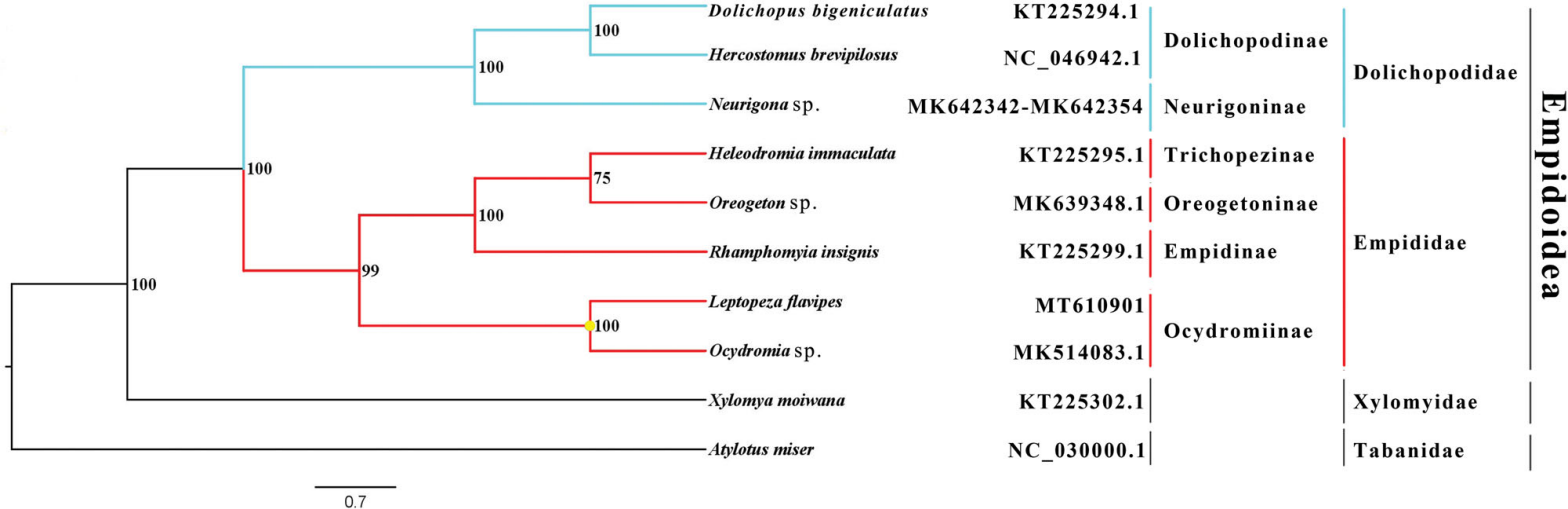
Bayesian phylogenetic tree of 10 Diptera species. The posterior probabilities are labeled at each node. Genbank accession numbers of all sequence used in the phylogenetic tree have been included in the figure and corresponding to the names of all species.

## Data Availability

The data that support the findings of this study are openly available in [NCBI] at [https://www.ncbi.nlm.nih.gov/], reference number [MT610901].
